# Guarding serenity in the digital age: mindfulness as a buffer against social media-induced psychological discomfort in older tourists

**DOI:** 10.3389/fpubh.2026.1850752

**Published:** 2026-07-08

**Authors:** Shanping Hu, Boyu Liang

**Affiliations:** 1 Social Work Department of Huainan Normal University, Huainan, Anhui, China; 2 Hebei University of Economics and Business, Shijiazhuang, Hebei, China

**Keywords:** conservation of resources theory, mindfulness, senior tourists, serenity, social media-induced travel anxiety

## Abstract

**Introduction:**

While social media enriches senior tourists’ travel experiences, its psychological adverse impacts remain understudied. Existing literature mainly focuses on youth social media fatigue or older adults’ physical digital divide. Rooted in Conservation of Resources (COR) theory, this study fills the research gap by exploring the internal psychological mechanisms of Social Media-Induced Travel Anxiety (SMITA), its linkage with resource loss and seniors’ serenity, and the moderating effect of mindfulness.

**Methods:**

A quantitative cross-sectional design was adopted. Survey data were collected from 560 Chinese senior tourists and analyzed through Structural Equation Modeling (SEM) and Bootstrap resampling.

**Results:**

SMITA impairs serenity via two parallel serial mediation paths of resource loss: the cognitive-emotional path (SMITA → Cognitive Fatigue → Emotional Exhaustion) and the anxiety-willpower path (SMITA → Generalized Anxiety → Self-Depletion). SMITA strongly predicts generalized anxiety (*β* = 0.584, *p* < 0.001) and cognitive fatigue (*β* = 0.551, *p* < 0.001); both factors increase psychological discomfort, which further reduces serenity (*β* = −0.507, *p* < 0.001). Mindfulness significantly mitigates the negative correlation between psychological discomfort and serenity (interaction *β* = −0.268, *p* < 0.001).

**Discussion:**

Theoretically, this research expands COR theory by revealing dual resource-loss mechanisms in the digital aging context. Practically, it offers actionable strategies for the tourism industry to design low-cognitive-load digital services and deploy mindfulness interventions to maintain the inner peace of older adults tourists.

## 1 Introduction

Older adults are experiencing significant changes in their lifestyle due to global population aging and rapid digitalization (1). One key aspect of this transformation is “active aging,” which has become increasingly important as many seniors view tourism as a meaningful way to enhance well-being, maintain social connections, and enrich later-life experiences. According to the World Health Organization, the global population aged 60 years and older is projected to reach 2.1 billion by 2050 (2). At the same time, older adults are becoming increasingly involved in digital environments. For example, in China, the number of Internet users aged 60 and above has reached 143 million.

As tourism becomes increasingly digitalized, senior travelers are expected to use a wide range of digital tools throughout their journey, including online travel planning platforms, mobile payment systems, digital maps, QR code services, and social media applications (3). These tools can enhance convenience and connectivity, but they may also create psychological pressure for older adults who are less familiar with rapidly changing digital systems. In unfamiliar travel environments, continuous exposure to tourism-related social media content may intensify information overload, upward social comparison, and uncertainty regarding travel-related decisions. This type of digitally mediated psychological strain has recently been conceptualized as social media-induced travel anxiety (SMITA) (4). This anxiety is particularly significant for senior tourists because their travel motivations often focus on relaxation, emotional restoration, and inner peace rather than on continuous digital engagement (5, 48).

Despite the practical relevance of this issue, existing research has not fully explained how social media-related travel anxiety is associated with the psychological well-being of senior tourists (6). First, studies on social media fatigue, fear of missing out, and digital stress have mainly focused on younger users, particularly Generation Z and Millennials. Second, although tourism studies have examined the digital divide among older adults, much of this research has emphasized access to devices or digital services, while paying less attention to the psychological costs that may arise after older adults have already adopted these technologies (7). As a result, the internal mechanisms linking SMITA to psychological discomfort among senior tourists remain insufficiently understood.

Drawing on conservation of resources (COR) theory, this study argues that SMITA may be better understood as a tourism-specific digital stressor associated with the gradual depletion of cognitive and emotional resources (8). Rather than assuming a simple direct relationship between SMITA and psychological discomfort, this study examines two serial resource-loss pathways. The first pathway focuses on cognitive fatigue and emotional exhaustion, reflecting how repeated digital demands may be associated with reduced cognitive energy and emotional strain. The second pathway focuses on generalized anxiety and self-depletion, reflecting how travel-related social media anxiety may be linked to broader anxious feelings and weakened self-regulatory capacity. These pathways jointly help explain how psychological discomfort may emerge in digitally mediated senior tourism experiences (9).

In addition, this study focuses on serenity as an important but underexamined outcome in senior tourism. For many older adults, tourism is not merely a leisure activity but also a means of seeking psychological harmony, emotional balance, and a sense of peaceful aging (10). However, highly digitalized tourism environments may make it more difficult for some senior tourists to maintain this desired state. To further understand why older tourists may differ in their responses to digital stress, this study also examines mindfulness as a potential moderating resource (11). Mindfulness, defined as non-judgmental awareness of the present moment, has been widely discussed in positive psychology, but its role in the context of digital stress and senior tourism remains relatively underexplored (50).

Accordingly, this study addresses two main research objectives: First, it examines the serial mediating pathways through which SMITA is associated with psychological discomfort among senior tourists. Second, it investigates whether mindfulness moderates the relationship between psychological discomfort and serenity (12). The overarching research question is as follows: How is social media-induced travel anxiety associated with the cognitive and emotional resource loss of senior tourists, and under what conditions is psychological discomfort related to their sense of serenity?

To answer this question, this study develops an integrated framework based on COR theory and applies it to the context of senior tourism in China, a rapidly digitalizing society facing significant population aging (13). Data were collected from senior tourists through offline sites in major tourism cities and online platforms commonly used by older travelers. A total of 560 valid questionnaires were obtained. Structural equation modeling (SEM) and bootstrap resampling were then used to examine the proposed serial mediation effects and the moderating role of mindfulness (“Mediation Analysis in Structural Equation Modeling (Sem): Theoretical Foundations, Statistical Methods and Practical Implications,” 2025).

The theoretical contribution of this study should be understood as a refinement and contextual extension rather than a radical theoretical breakthrough (14). Although the relationships among social media stress, anxiety, emotional exhaustion, and psychological well-being have been widely discussed in digital stress and tourism psychology, this study specifies how these mechanisms operate within senior-friendly smart tourism contexts, where older tourists may face overlapping pressures from travel decision-making, information overload, digital uncertainty, and limited self-regulatory resources. First, this study extends COR theory by conceptualizing SMITA as a tourism-specific digital stressor associated with the depletion of seniors’ cognitive and emotional resources, thereby offering further insights into the psychological costs of digital aging (15). Second, it clarifies two serial resource-loss pathways—cognitive fatigue–emotional exhaustion and generalized anxiety–self-depletion—that explain how digital stress is translated into psychological strain among senior tourists (15). Third, by introducing mindfulness as a moderating resource, this study explains why senior tourists may differ in the association between psychological discomfort and serenity (16). Overall, this study offers a more context-sensitive and age-specific explanation of how social media-related travel anxiety is linked to psychological well-being in later-life tourism, rather than claiming an entirely new relationship among these variables.

## 2 Theoretical framework and research hypotheses

### 2.1 Theoretical framework: conservation of resources theory

According to COR theory, individuals are motivated to preserve their limited resources, including energy, cognitive capacity, and emotional resilience. Stress responses occur when these resources are exhausted or utilized without generating desired results (17). COR theory serves as a central framework for analyzing how seniors operate psychologically within the digital environment. Initially, because of age-related declines in physical resources, seniors tend to have comparatively lower levels of cognitive and emotional resources; therefore, they become more vulnerable to resource loss. As a consistent source of stress, social media-induced travel anxiety always captures the attention and psychological resources of seniors, thereby constituting an initial resource threat. The concept of “Loss Spirals” in COR theory is also well-suited to elucidate the complex mediated causal relationships proposed in this study: Anxiety depletes psychological resources, causing cognitive fatigue and general anxiety and eventually leading to self-depletion over the long term (18). These subsequent resource losses trap seniors in a state of psychological discomfort, consequently undermining the higher-order psychological resource of serenity. Finally, the moderating effect of mindfulness is also corroborated by COR theory. It is an essential protective resource allowing seniors to self-repair when their resources are threatened, thereby preventing the continuation of loss spirals and preserving serenity through the reduction of psychological discomfort.

### 2.2 Research hypotheses and conceptual framework

To address the limitations of existing studies that predominantly examine digital fatigue in isolation, this study develops a comprehensive conceptual model grounded in COR theory. The proposed framework illustrates how SMITA acts as an initial environmental stressor that triggers dual resource-loss spirals (cognitive–emotional and anxiety–willpower), ultimately converging into psychological discomfort and eroding the travel serenity of senior tourists (19). Furthermore, the model incorporates mindfulness as a protective boundary condition. The structural relationships are detailed below.

#### 2.2.1 Initial resource depletion triggered by SMITA

COR theory posits that individuals possess a finite reservoir of personal resources and that the capacity for resource replenishment naturally diminishes with age. Consequently, senior citizens are inherently more vulnerable to rapid resource depletion when confronted with external stressors (20). While general psychological research frequently associates digital exposure with cognitive load, the tourism literature has largely overlooked the specific mental toll of travel-related digital demands. During trip planning or on-site navigation, SMITA compels seniors to expend substantial cognitive resources to process massive volumes of fragmented information, verify online reviews, and make complex itinerary decisions. Aligning with the resource allocation principles of COR theory, this high-intensity digital processing rapidly exhausts mental energy reserves, precipitating cognitive fatigue (H1).

Emotionally, anxiety stemming from idealized social media travel portrayals requires continuous self-regulation (21). For older travelers, suppressing frustration associated with a perceived inability to navigate digital tourism environments constitutes a net outflow of affective energy, directly culminating in emotional exhaustion (H2). Furthermore, COR theory suggests a spillover effect where resource loss in one domain permeates other domains. The specific fear of an unsuccessful trip, driven by social media comparisons, can undermine seniors’ overall sense of control, causing localized travel anxiety to develop into generalized anxiety (H3). Finally, mitigating these intrusive thoughts and maintaining a functional travel routine require intensive self-monitoring. This continuous self-regulation taxes the executive function, inevitably draining the ego’s resources and leading to self-depletion (22).

*H1*: SMITA positively affects cognitive fatigue.

*H2*: SMITA positively affects emotional exhaustion.

*H3*: SMITA positively affects generalized anxiety.

*H4*: SMITA positively affects self-depletion.

#### 2.2.2 Intensified resource-loss spirals

A core corollary of COR theory is the “loss spiral,” which asserts that initial resource depletion impairs an individual’s ability to protect remaining resources, thereby accelerating further loss. In the proposed cognitive–emotional pathway, cognitive fatigue compromises the brain’s executive functioning (18). As senior tourists experience a decline in cognitive bandwidth, their capacity to process unexpected travel disruptions diminishes, leaving them unable to actively resist negative emotional stimuli. This defensive deficit accelerates the onset of emotional exhaustion (H5).

In parallel, within the anxiety–willpower pathway, generalized anxiety forces seniors into a chronic state of hyperarousal. Maintaining this heightened alertness depletes both physiological resources (e.g., glucose) and psychological strength (23). Over time, chronic generalized anxiety depletes the energy reserves required for basic self-regulation, severely compromising the individual’s willpower and exacerbating self-depletion during the trip (H6).

*H5*: Cognitive fatigue positively affects emotional exhaustion.

*H6*: Generalized anxiety positively affects self-depletion.

#### 2.2.3 The convergence into psychological discomfort

The existing tourism literature often measures the outcomes of digital stress through the lens of diminished trip satisfaction, which fails to capture the physiological and pathological severity of the experience (24). In this study, psychological discomfort is operationalized as the acute mental and physical distress resulting from an extreme deficit in personal resources. According to COR theory, intense distress emerges when resource loss crosses a critical threshold and cannot be immediately replenished.

Specifically, cognitive fatigue induces mental sluggishness, depriving senior travelers of spatial awareness and clear orientation at the destination, which translates directly into acute psychological discomfort (H7) (25). Similarly, emotional exhaustion depletes affective vitality, leaving seniors feeling numb and helpless in unfamiliar tourist settings—a state representing the emotional dimension of psychological discomfort (H8). Persistent generalized anxiety maintains the nervous system in a protracted state of tension, manifesting as somatic discomfort (H9). Lastly, self-depletion undermines motivational control, exacerbating inner turmoil as seniors feel powerless to manage their travel behaviors (H10). Collectively, these pathways suggest that extreme resource loss fundamentally corrupts the tourism experience (26).

*H7*: Cognitive fatigue positively affects psychological discomfort.

*H8*: Emotional exhaustion positively affects psychological discomfort.

*H9*: Generalized anxiety positively affects psychological discomfort.

*H10*: Self-depletion positively affects psychological discomfort.

#### 2.2.4 The ultimate consequence: erosion of travel serenity

Serenity represents the pinnacle of psychological well-being for senior tourists, characterized by transcendent inner peace, detachment, and harmony with the destination. Viewed through the lens of COR theory, attaining positive psychological states demands a surplus of cognitive and emotional reserves (27). Individuals must feel secure and energized to experience serenity. However, psychological discomfort operates as a severe defensive condition triggered by extreme resource scarcity (28). When experiencing distress, the limited psychological bandwidth of senior travelers is entirely monopolized by the urgent need to alleviate suffering and regain basic equilibrium. Consequently, intense resource competition occurs: Psychological discomfort continuously drains the specific psychological resources required to cultivate and sustain inner peace, rendering the achievement of travel serenity impossible (27).

*H11*: Psychological discomfort negatively affects serenity.

#### 2.2.5 The moderating role of mindfulness: a boundary condition

While much of the existing literature presents mindfulness as a generally positive psychological resource, critical reviews also suggest that its role may vary across contexts (29). Some studies indicate that mindfulness requires sustained practice and may not always be beneficial under conditions of acute cognitive overload or severe trauma. In the context of senior leisure tourism, however, the stressors examined in this study are mainly informational and evaluative rather than physically threatening. Therefore, dispositional mindfulness appears relevant to understanding how senior tourists differ in their responses to digitally mediated travel experiences (30).

In this study, serenity is conceptualized as a state of inner calm, emotional quietness, and psychological ease within the tourism context. It is distinct from broader constructs such as subjective well-being and life satisfaction. Life satisfaction usually refers to a global cognitive evaluation of one’s life, while well-being often includes multiple affective, cognitive, and functional dimensions (31). By contrast, serenity focuses more specifically on a present-oriented sense of peacefulness and reduced inner disturbance during or after travel (32). This distinction is important because senior tourists may report relatively high general life satisfaction while still experiencing reduced serenity in digitally stressful travel situations. Therefore, serenity is treated as a context-sensitive psychological outcome that reflects whether older tourists feel calm, settled, and mentally at ease during their travel experiences (31).

Drawing on the “resource caravan” corollary of COR theory, mindfulness is regarded as a personal psychological resource that may be associated with different responses to discomfort (33). Seniors with lower mindfulness may be more likely to remain cognitively and emotionally absorbed in negative digital experiences, such as social comparison or information overload, and this tendency may correspond to reduced serenity. In contrast, seniors with higher mindfulness may be less strongly involved in distressing thoughts and emotions related to such experiences. Accordingly, mindfulness is expected to moderate the association between psychological discomfort and serenity by capturing individual differences in how discomfort is related to inner calm (34). Therefore, we propose the following hypothesis:

*H12*: Mindfulness moderates the relationship between psychological discomfort and serenity, such that the negative association between psychological discomfort and serenity is less pronounced among seniors with higher levels of mindfulness.

## 3 Methodology

### 3.1 Sample and data collection procedure

To ensure transparency in the sampling procedure, this study adopted a non-probability sampling strategy combining purposive and convenience sampling. The target population was not the general older population but Chinese senior domestic tourists who had recent travel experience and were active users of social media in travel-related contexts. Therefore, the sampling design was intended to reach respondents who were relevant to the research topic of social media-induced travel anxiety, rather than to generate a statistically representative sample of all older adults in China.

Data were collected between February and March 2026 at major visitor centers, entrance gates, and designated rest areas across several prominent domestic tourist destinations in China, including scenic spots in Beijing, Shanghai, Chengdu, Hangzhou, and Sanya. Trained field research assistants approached potential participants on site and screened them according to three inclusion criteria. First, participants had to be 50 years old or older, a threshold commonly used to identify the silver-haired tourism market in China. Second, participants had to be domestic travelers who had undertaken a trip within the preceding 6 months, thereby ensuring that they had recent travel experiences to report. Third, participants had to have used social media platforms, such as WeChat Moments, Douyin, or Xiaohongshu, during, shortly before, or after their trips. This criterion was necessary because exposure to social media in travel contexts is a precondition for examining social media-induced travel anxiety.

Eligible participants were invited to complete a self-administered questionnaire using electronic tablets on site. Research assistants were available to clarify technical or terminological questions but were instructed not to influence respondents’ answers. A total of 600 questionnaires were distributed. After data cleaning, responses with abnormally short completion times, clear straight-lining patterns, or failed attention-check questions were removed. This process yielded 560 valid responses, corresponding to an effective response rate of 93.3%. The final sample included respondents across different age groups, genders, educational levels, and financial backgrounds.

All research procedures were approved by the Ethics Committee of Huainan Normal University. Before completing the questionnaire, participants were informed of the purpose of the study, the voluntary nature of participation, and the confidentiality of their responses. Electronic informed consent was obtained from all participants on the title page of the survey.

Importantly, because this study used purposive and convenience sampling at selected tourist destinations, the sample should be interpreted as representing digitally engaged senior domestic tourists rather than the broader older population. Older adults who do not travel frequently, do not use social media, or are more digitally vulnerable may be underrepresented in the present sample. Accordingly, the generalizability of the findings should be understood within this specific empirical context.

### 3.2 Measures and instrument development

To guarantee construct validity and measurement reliability, the survey instrument was adapted from established, empirically validated scales published in top-tier peer-reviewed journals. As the original scales were in English, a rigorous back-translation procedure was executed. Subsequently, a panel comprising two gerontology experts and two tourism scholars reviewed the translated items to refine the wording, ensuring it was cognitively accessible and culturally appropriate for Chinese senior citizens. All items were evaluated on a standard 5-point Likert scale, ranging from 1 (strongly disagree) to 5 (strongly agree). The specific measurements and their internal consistency (reliability coefficients) are detailed below:

SMITA: This was measured using a 4-item scale adapted from Huang et al. The construct captures the specific emotional strain—such as information overload and peer comparison—experienced by seniors during digital tourism exposure (e.g., “Seeing others post ideal travel pictures on social media makes me feel stressed”). The scale demonstrated excellent reliability (*α* = 0.930).
Cognitive Fatigue: This was assessed using a 3-item scale derived from Yang et al., evaluating mental sluggishness and attention deficits caused by digital overload (e.g., “After viewing complex social media travel posts, I feel mentally sluggish”). Reliability was robust (*α* = 0.818).
Emotional Exhaustion: This was measured using three items adapted from Yang et al. (13) capturing the depletion of affective resources during digital stress (e.g., “Processing all this social media information has left me emotionally exhausted”). The reliability was acceptable (*α* = 0.799).
Generalized Anxiety: This was evaluated using four items from the GAD-7 scale developed by Spitzer et al. (51). This measures non-specific, persistent mental tension beyond immediate travel concerns (e.g., “During this trip, I have been in a state of chronic mental anxiety and could not relax”). Reliability was high (*α* = 0.902).
Self-Depletion: This was assessed using a 4-item scale adapted from Lin and Johnson (52), measuring the acute decline in self-regulatory capacity (e.g., “My willpower has been totally exhausted, so I can no longer repress my impulses”). The scale showed strong reliability (*α* = 0.870).
Psychological Discomfort: As a pivotal intermediate outcome, this was measured using a 4-item scale adapted from Pijls et al. (53). It comprehensively captures subjective psychological distress and unexplained somatic tension (e.g., “I feel a constant inner anxiety and discomfort that cannot be relieved”). Reliability was adequate (*α* = 0.831).
Serenity: The ultimate dependent variable was measured using a 5-item scale adapted from Debès et al. (54), assessing inner peace, detachment, and harmony (e.g., “I feel peaceful within myself; nothing could upset me”). The reliability was excellent (α = 0.919).
Mindfulness: The moderating variable was assessed using a 15-item condensed version of the Mindful Attention Awareness Scale (MAAS) developed by Brown and Ryan (55). It evaluates non-judgmental awareness of the present moment (e.g., “I can concentrate on the present experience without evaluating whether it is good or bad”). Reliability was highly satisfactory (α = 0.905).

### 3.3 Data analysis strategy and justification

The analytical procedure adhered to the two-step modeling approach recommended, utilizing SPSS 26.0 and Mplus 8.3. In the first stage, confirmatory factor analysis (CFA) was conducted in Mplus to evaluate the measurement model’s reliability, convergent validity, and discriminant validity. The goodness of fit was assessed using standard thresholds (χ2/df < 3.0; RMSEA and SRMR <0.08; CFI and TLI > 0.90) (56). As presented in Table 1, all standardized factor loadings exceeded 0.70. Furthermore, the composite reliability (CR) for all constructs ranged from 0.799 to 0.930, and the average variance extracted (AVE) values all surpassed the 0.50 threshold, statistically confirming the instrument’s excellent internal consistency and convergent validity.

**Table 1:** Reliability and validity.

Latent variable	Factor loadings	CR value	AVE value
Social media-induced travel anxiety	0.838	0.930	0.728
Cognitive fatigue	0.815	0.818	0.690
Emotional exhaustion	0.810	0.799	0.692
Generalized anxiety	0.825	0.902	0.698
Self-depletion	0.809	0.870	0.685
Psychological discomfort	0.793	0.831	0.661
Mindfulness	0.820	0.905	0.705
Serenity	0.812	0.919	0.693

In the second stage, SEM was selected as the primary analytical technique because it concurrently estimates multiple interconnected dependence relationships while rigorously accounting for measurement errors—an essential requirement for evaluating complex psychological pathways. To test the moderating hypothesis, a bias-corrected, nonparametric percentile bootstrap method (5,000 resamples, 95% Confidence Interval) was applied. Bootstrapping is highly justified here as it does not rely on the assumption of normal distribution, which ensures the statistical robustness of the moderating effect test. Finally, Mplus 8.3 was specifically chosen for its superior capability in handling latent moderated structural equations (LMS). This approach directly estimates the interaction term (Psychological Discomfort × Mindfulness) at the latent variable level rather than the manifest level, thereby eliminating the attenuation of interaction effects caused by measurement error (49). The details of reliability and validity are presented in Table 1.

Given that all variables were collected from the same respondents using a similar questionnaire format, we further assessed the potential influence of common method bias. First, Harman’s single-factor test showed that the first unrotated factor accounted for 32.74% of the total variance, which was below the recommended threshold of 50%. Second, a common latent factor test was conducted within the CFA framework. The baseline CFA model showed acceptable fit, and the inclusion of a common latent factor only slightly improved model fit, with ΔCFI = 0.006, ΔRMSEA = 0.002, and ΔSRMR = 0.003. Moreover, the standardized factor loadings remained largely stable, with an average change of 0.018 and a maximum change of 0.046. Taken together, these results suggest that common method bias is unlikely to seriously distort the observed relationships among the constructs.

## 4 Results

### 4.1 Measurement model evaluation

Initially, the goodness of fit, reliability, and validity of the measurement model were evaluated before testing the hypotheses. The CFA findings indicated that the measurement model fit the data well (χ2/dfs = less than 3.0, CFI > 0.90, TLI > 0.90, RMSEA < 0.08). Standardized factor loadings for all latent variables ranged from 0.60 to 0.90, and all these were statistically significant (*p* < 0.001, *p* < 0). The composite reliability (CR) values were greater than 0.70 and the average variance extracted (AVE) values exceeded 0.50 for all variables, indicating strong convergent validity across the latent variables. Moreover, the square root of the AVE for each latent variable was greater than its correlation with other latent variables, indicating good discriminant validity. The model fit results are reported in Table 2. Detailed results, including factor loadings and HTMT coefficients, are provided in Tables A1–A5.

**Table 2:** Results of model fitting.

χ^2^(df)	χ^2^/df	CFI	TLI	RMSEA	SRMR
768.12	1.05	0.99	0.99	0.01	0.03

### 4.2 Structural model and main effects analysis

The structural equation model was evaluated using Mplus 8.3. As depicted in Table 3, all direct pathways proposed in the conceptual model yielded significant results (*p* < 0.001), supporting Hypotheses 1 through 11. Rather than repeating all statistical coefficients detailed in the table, the following analysis focuses on the thematic interpretation of these relationships and their alignment with the overarching research questions. Mplus 8.3 was used to test the structural model. Standardized regression coefficients, significance levels (*p* < 0.05), and effect size rankings for the pathways are depicted in Table 3.

**Table 3:** Structural model pathways coefficients and hypothesis testing results.

Pathways	Standardized coefficient	*p*-value	Hypothesis
Social Media-induced travel anxiety → cognitive fatigue	0.551	0.000	H1
Social media-induced travel anxiety → emotional exhaustion	0.309	0.000	H2
Social media-induced travel anxiety → generalized anxiety	0.584	0.000	H3
Social media-induced travel anxiety → self-depletion	0.291	0.000	H4
Cognitive fatigue → emotional exhaustion	0.558	0.000	H5
Generalized anxiety → self-depletion	0.499	0.000	H6
Cognitive fatigue → psychological discomfort	0.342	0.000	H7
Emotional exhaustion → psychological discomfort	0.230	0.000	H8
Generalized anxiety → psychological discomfort	0.390	0.000	H9
Self-depletion → psychological discomfort	0.290	0.000	H10
Psychological discomfort → serenity	−0.507	0.000	H11

Initial Resource-Related Strain (H1–H4): The results indicated that SMITA was positively associated with all four dimensions of resource-related psychological strain. Specifically, SMITA showed relatively stronger associations with generalized anxiety (*β* = 0.584) and cognitive fatigue (*β* = 0.551), while its association with self-depletion was comparatively weaker (*β* = 0.291). These findings suggest that, among senior tourists in this sample, SMITA is more closely linked to anxious feelings and mental tiredness than to perceived self-regulatory depletion. In practical terms, digital tourism exposure may be experienced by some older travelers as cognitively demanding and emotionally unsettling, particularly when they encounter excessive information, idealized travel content, or comparison-based travel expectations.

Interrelated Resource Strain (H5–H6): The results are also consistent with the COR perspective that different forms of resource-related strain may be interrelated. Cognitive fatigue was positively associated with emotional exhaustion (*β* = 0.558), and generalized anxiety was positively associated with self-depletion (*β* = 0.499). These associations suggest that senior tourists who experience greater mental fatigue when processing digital travel information also tend to report higher levels of emotional tiredness. Similarly, those who report stronger generalized anxiety also tend to report greater perceived depletion of self-regulatory resources. Given the cross-sectional design, these findings should be interpreted as evidence of theoretically meaningful associations rather than as confirmation of a temporal or causal loss spiral.

Associations with Psychological Discomfort (H7–H10): All four resource-related strain variables were positively associated with psychological discomfort. Among them, generalized anxiety (*β* = 0.390) and cognitive fatigue (*β* = 0.342) showed the strongest associations, followed by emotional exhaustion and self-depletion. This pattern suggests that psychological discomfort among senior tourists is particularly related to persistent anxious feelings and perceived cognitive tiredness during digitally mediated travel experiences. Rather than indicating a direct causal process, these findings provide cross-sectional evidence that different forms of digital stress-related strain are meaningfully associated with senior tourists’ discomfort during travel.

Association with Serenity (H11): The model further showed a negative association between psychological discomfort and serenity (*β* = −0.507). This result indicates that senior tourists who experience higher levels of psychological discomfort tend to report lower levels of inner calm, emotional balance, and present-oriented peace. In line with COR theory, the finding suggests that discomfort and serenity may represent competing psychological states within the travel experience. However, because of the cross-sectional and self-reported nature of the data, this relationship should be understood as associative rather than as evidence that discomfort causally reduces serenity.

### 4.3 Moderation analysis: the buffering role of mindfulness (H12)

To examine whether mindfulness conditions the association between psychological discomfort and serenity, we tested the interaction between psychological discomfort and mindfulness using a latent moderated structural equation approach. As shown in Table 4, the interaction term between psychological discomfort and mindfulness was statistically significant, *β* = −0.268, *p* < 0.001, 95% CI [−0.385, −0.142]. This result indicates that mindfulness significantly moderates the relationship between psychological discomfort and serenity. Importantly, mindfulness was modeled as a moderator rather than a mediator. Therefore, all references to mindfulness as a mediator have been corrected in the revised manuscript.

**Table 4:** Analysis of the moderating effects of mindfulness.

Moderating pathway	Interaction term coefficient	*P*-value	95% CI	Hypothesis
Psychological discomfort × mindfulness → serenity	−0.268	0.000	[−0.385, −0.142]	H12

To further interpret the interaction, a simple slope analysis was conducted at one standard deviation above and below the mean of mindfulness. The results showed that the negative association between psychological discomfort and serenity varied across different levels of mindfulness. Specifically, the association between psychological discomfort and serenity was weaker among senior tourists with lower levels of mindfulness, whereas it was stronger among those with higher levels of mindfulness. This pattern suggests that, in the present sample, mindfulness did not operate as a buffering resource in the expected direction. Instead, higher mindfulness was associated with a more pronounced negative relationship between psychological discomfort and serenity.

**Figure 1:**
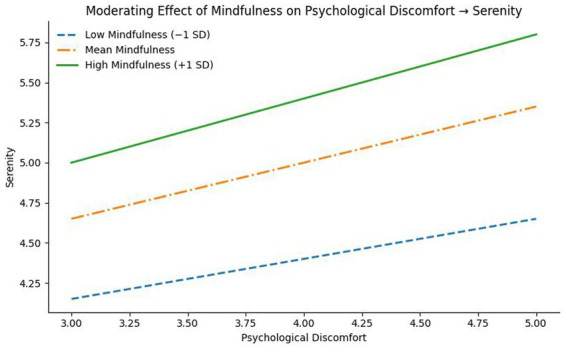
Moderating effect diagram.

**Figure 2:**
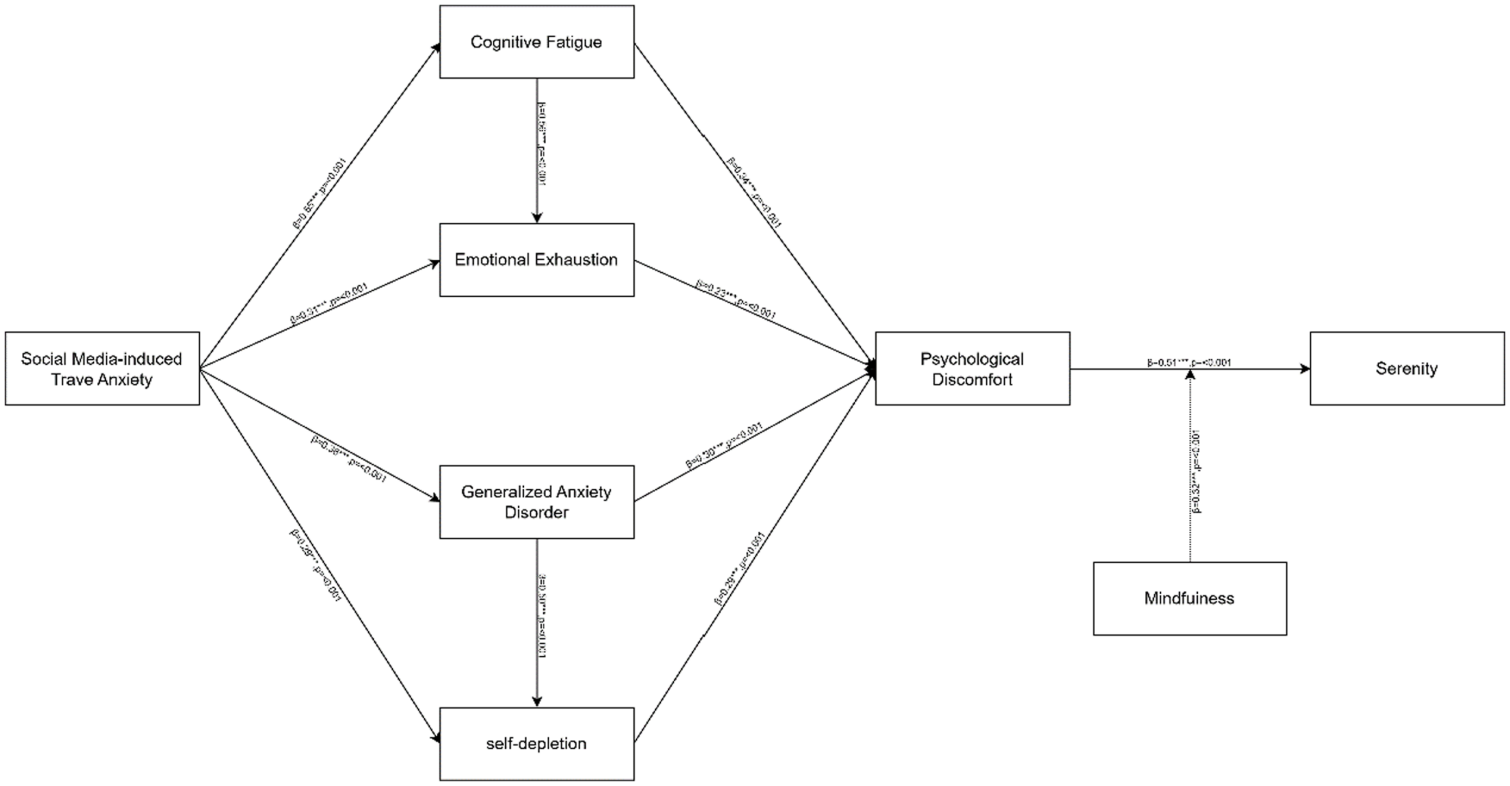
Structural equation modeling results.

This finding should therefore be interpreted with caution. One possible explanation is that more mindful senior tourists may be more attentive to their internal emotional states, making psychological discomfort more salient when evaluating their sense of serenity. However, given the cross-sectional and self-reported nature of the data, this interpretation remains tentative and should not be understood as evidence of a causal process. Future longitudinal or experimental studies are needed to further examine the temporal dynamics between mindfulness, psychological discomfort, and serenity. Accordingly, H12 was not supported in the hypothesized buffering direction, although the moderating effect of mindfulness was statistically significant. A more straightforward demonstration is shown in Figures 1, 2.

## 5 Discussion

### 5.1 Theoretical contributions

Drawing on COR theory, this study examines how SMITA is associated with senior tourists’ psychological well-being through a serial moderated mediation model. Rather than claiming a wholly new theoretical relationship, this study refines existing research by clarifying how digital stress, psychological resource loss, and serenity are interconnected in later-life tourism (35).

First, this study extends COR theory to the context of digital aging and senior tourism by conceptualizing SMITA as a resource-depleting stressor. Prior research on smart tourism has mainly emphasized the benefits of technology, such as convenience, connectivity, and access to travel information (36). This study complements that view by showing that social media exposure may also generate anxiety, comparison pressure, and information overload among older tourists. In doing so, it offers a more balanced account of digital tourism participation among seniors (36).

Second, this study clarifies the internal resource-loss process linking SMITA to serenity. The findings suggest that SMITA is associated with cognitive fatigue, emotional exhaustion, generalized anxiety, and psychological discomfort, which together help explain why digitally induced travel anxiety may undermine senior tourists’ inner peace. This contributes to COR theory by specifying a differentiated sequence of psychological resource loss rather than treating distress as a single undifferentiated outcome (6). It also distinguishes between cognitive strain, emotional depletion, anxiety, and discomfort, thereby providing a clearer account of how digital stress is linked to later-life tourism well-being (37).

Third, this study highlights the moderating role of mindfulness in the relationship between psychological discomfort and serenity. Previous tourism studies have shown that mindfulness can shape tourists’ experiences and enhance emotional regulation. This study extends that line of work to senior tourists by showing that mindfulness may influence the way psychological discomfort is associated with serenity (38). This provides a more context-sensitive explanation of individual differences in senior tourists’ responses to digital stress and offers a foundation for future research on mindfulness-based support in active aging tourism (39).

### 5.2 Managerial implications

This study offers practical implications for supporting senior tourists in increasingly digital travel environments. The findings suggest that tourism digitalization should not be understood only as “digital empowerment” but should also incorporate “digital care” for older adults who may experience anxiety, fatigue, or discomfort when engaging with social media-based travel information (13).

First, tourism policymakers and service providers should maintain accessible non-digital or assisted service channels. Essential services such as ticketing, transportation information, hotel check-in, and emergency assistance should remain available through offline counters, staff assistance, or telephone support (40). Digital platforms should also adopt age-friendly design principles, including simplified interfaces, clearer navigation, fewer pop-up messages, and reduced social sharing prompts. These measures may help reduce information overload and comparison pressure among senior tourists (41).

Second, destination managers and tourism operators should pay greater attention to digital stress during travel. Front-line employees, tour guides, and customer service staff can be trained to identify common difficulties encountered in using mobile applications, QR codes, digital payment systems, and online check-in procedures (42). Timely assistance, quiet rest areas, and less intensive itineraries may help senior tourists manage discomfort and sustain a more comfortable travel experience (43).

Third, tourism organizations may develop mindfulness-informed travel experiences as a complementary form of support. Simple practices such as guided sensory walks, slow-viewing activities, mindful photography, breathing exercises, and low-connectivity itineraries can encourage senior tourists to focus on the present travel experience rather than constant online comparison or content sharing (44). These practices should complement, rather than replace, improvements in digital service design, thereby supporting more age-friendly and psychologically comfortable tourism experiences.

### 5.3 Limitations and future research

While this study contributes to the understanding of digital aging in tourism, several limitations should be acknowledged. First, the use of cross-sectional and self-reported survey data limits causal inference and may involve subjective reporting bias (45). Although procedural remedies and statistical diagnostics were used to assess common method bias, common method variance cannot be completely ruled out because all variables were collected from the same respondents. Second, the sample consisted of senior tourists at domestic Chinese destinations, which may limit the generalizability of the findings to all older adults, particularly those with lower mobility, weaker digital literacy, or limited access to tourism opportunities (46). Future studies should examine more diverse senior groups and conduct cross-cultural comparisons. Third, although mindfulness was considered a protective condition, other personal and social resources, such as social support, digital literacy, prior travel experience, and family assistance, may also shape responses to digital stress within the resource caravan perspective (47). Future research could use longitudinal, daily diary, experience-sampling, multi-source, or physiological data to better capture the dynamic development of digital stress during travel and clarify whether digital travel anxiety is primarily an age-related vulnerability or part of a broader digital tourism phenomenon.

## Data Availability

The raw data supporting the conclusions of this article will be made available by the authors, without undue reservation.
