# Supplementary material for: Guarding serenity in the digital age: mindfulness as a buffer against social media-induced psychological discomfort in older tourists
Source: Front Public Health. 2026 Jul 8;14:1850752. doi: 10.3389/fpubh.2026.1850752 (PMC13388086; doi:10.3389/fpubh.2026.1850752)
Supplement: Supplementary file 1 [file Table_1.docx]

**Table A1. Standardized Factor Loadings, Cronbach’s Alpha, CR, and AVE**

| **Construct** | **Item** | **Standardized Loading** | **Cronbach’s α** | **CR** | **AVE** |
| --- | --- | --- | --- | --- | --- |
| SMITA | SMITA1 | 0.88 | 0.930 | 0.94 | 0.80 |
|  | SMITA2 | 0.91 |  |  |  |
|  | SMITA3 | 0.89 |  |  |  |
|  | SMITA4 | 0.90 |  |  |  |
| Cognitive Fatigue | CF1 | 0.78 | 0.818 | 0.83 | 0.62 |
|  | CF2 | 0.81 |  |  |  |
|  | CF3 | 0.77 |  |  |  |
| Emotional Exhaustion | EE1 | 0.76 | 0.799 | 0.82 | 0.60 |
|  | EE2 | 0.79 |  |  |  |
|  | EE3 | 0.77 |  |  |  |
| Generalized Anxiety | GA1 | 0.84 | 0.902 | 0.92 | 0.73 |
|  | GA2 | 0.87 |  |  |  |
|  | GA3 | 0.86 |  |  |  |
|  | GA4 | 0.85 |  |  |  |
| Self-Depletion | SD1 | 0.79 | 0.870 | 0.89 | 0.67 |
|  | SD2 | 0.84 |  |  |  |
|  | SD3 | 0.82 |  |  |  |
|  | SD4 | 0.83 |  |  |  |
| Psychological Discomfort | PD1 | 0.76 | 0.831 | 0.86 | 0.61 |
|  | PD2 | 0.80 |  |  |  |
|  | PD3 | 0.78 |  |  |  |
|  | PD4 | 0.79 |  |  |  |
| Serenity | SER1 | 0.85 | 0.919 | 0.93 | 0.72 |
|  | SER2 | 0.87 |  |  |  |
|  | SER3 | 0.86 |  |  |  |
|  | SER4 | 0.84 |  |  |  |
|  | SER5 | 0.82 |  |  |  |
| Mindfulness | MIN1 | 0.69 | 0.905 | 0.92 | 0.51 |
|  | MIN2 | 0.72 |  |  |  |
|  | MIN3 | 0.70 |  |  |  |
|  | MIN4 | 0.68 |  |  |  |
|  | MIN5 | 0.74 |  |  |  |
|  | MIN6 | 0.71 |  |  |  |
|  | MIN7 | 0.73 |  |  |  |
|  | MIN8 | 0.67 |  |  |  |
|  | MIN9 | 0.75 |  |  |  |
|  | MIN10 | 0.70 |  |  |  |
|  | MIN11 | 0.66 |  |  |  |
|  | MIN12 | 0.72 |  |  |  |
|  | MIN13 | 0.69 |  |  |  |
|  | MIN14 | 0.71 |  |  |  |
|  | MIN15 | 0.68 |  |  |  |

Table A2. Corrected Fornell–Larcker Discriminant Validity Matrix

| **Construct** | **SMITA** | **CF** | **EE** | **GA** | **SD** | **PD** | **SER** | **MIN** |
| --- | --- | --- | --- | --- | --- | --- | --- | --- |
| SMITA | **0.894** |  |  |  |  |  |  |  |
| CF | 0.56 | **0.787** |  |  |  |  |  |  |
| EE | 0.58 | 0.61 | **0.775** |  |  |  |  |  |
| GA | 0.62 | 0.57 | 0.60 | **0.854** |  |  |  |  |
| SD | 0.47 | 0.52 | 0.54 | 0.53 | **0.819** |  |  |  |
| PD | 0.60 | 0.58 | 0.64 | 0.69 | 0.56 | **0.781** |  |  |
| SER | -0.43 | -0.39 | -0.44 | -0.48 | -0.37 | -0.53 | **0.849** |  |
| MIN | -0.30 | -0.27 | -0.29 | -0.33 | -0.25 | -0.35 | 0.41 | **0.714** |

TableA 3. HTMT Matrix of Study Constructs

| **Construct** | **SMITA** | **CF** | **EE** | **GA** | **SD** | **PD** | **MF** | **SE** |
| --- | --- | --- | --- | --- | --- | --- | --- | --- |
| SMITA | — |  |  |  |  |  |  |  |
| CF | 0.68 | — |  |  |  |  |  |  |
| EE | 0.55 | 0.72 | — |  |  |  |  |  |
| GA | 0.71 | 0.64 | 0.66 | — |  |  |  |  |
| SD | 0.50 | 0.57 | 0.61 | 0.69 | — |  |  |  |
| PD | 0.63 | 0.70 | 0.68 | 0.76 | 0.65 | — |  |  |
| MF | 0.31 | 0.28 | 0.34 | 0.36 | 0.32 | 0.39 | — |  |
| SE | 0.42 | 0.45 | 0.47 | 0.50 | 0.43 | 0.62 | 0.48 | — |

Note: SMITA = Social Media-Induced Travel Anxiety; CF = Cognitive Fatigue; EE = Emotional Exhaustion; GA = Generalized Anxiety; SD = Self-depletion; PD = Psychological Discomfort; MF = Mindfulness; SE = Serenity.

Table A4. Cross-Loading Diagnostic Summary

| **Construct** | **Number of Items** | **Primary Loading Range** | **Highest Cross-Loading Range** | **Evaluation** |
| --- | --- | --- | --- | --- |
| SMITA | 4 | 0.88–0.91 | 0.32–0.45 | Acceptable |
| Cognitive Fatigue | 3 | 0.77–0.81 | 0.30–0.43 | Acceptable |
| Emotional Exhaustion | 3 | 0.76–0.79 | 0.34–0.47 | Acceptable |
| Generalized Anxiety | 4 | 0.84–0.87 | 0.36–0.52 | Acceptable |
| Self-Depletion | 4 | 0.79–0.84 | 0.28–0.43 | Acceptable |
| Psychological Discomfort | 4 | 0.76–0.80 | 0.39–0.55 | Acceptable |
| Serenity | 5 | 0.82–0.87 | 0.31–0.46 | Acceptable |
| Mindfulness | 15 | 0.66–0.75 | 0.18–0.36 | Acceptable |

Table A5. Corrected Competing CFA Model Comparison

| **Model** | **Model Specification** | **χ²** | **df** | **χ²/df** | **CFI** | **TLI** | **RMSEA** | **SRMR** |
| --- | --- | --- | --- | --- | --- | --- | --- | --- |
| M1 | Eight-factor model: all constructs separate | 1435.28 | 791 | 1.81 | 0.956 | 0.952 | 0.043 | 0.040 |
| M2 | Seven-factor model: GA and PD combined | 1768.43 | 798 | 2.22 | 0.934 | 0.928 | 0.053 | 0.055 |
| M3 | Seven-factor model: CF and EE combined | 1692.76 | 798 | 2.12 | 0.939 | 0.933 | 0.051 | 0.052 |
| M4 | Six-factor model: CF, EE, and GA combined | 2045.91 | 804 | 2.54 | 0.916 | 0.907 | 0.061 | 0.067 |
| M5 | Five-factor model: CF, EE, GA, and PD combined | 2387.64 | 809 | 2.95 | 0.893 | 0.881 | 0.069 | 0.076 |
| M6 | One-factor model: all items loaded on one factor | 4062.18 | 819 | 4.96 | 0.781 | 0.765 | 0.105 | 0.109 |
